# Using activity theory to study cultural complexity in medical education

**DOI:** 10.1007/s40037-014-0114-3

**Published:** 2014-03-04

**Authors:** Janneke M. Frambach, Erik W. Driessen, Cees P. M. van der Vleuten

**Affiliations:** Department of Educational Development and Research, Faculty of Health, Medicine and Life Sciences, Maastricht University, PO Box 616, 6200 MD Maastricht, The Netherlands

**Keywords:** Activity theory, Socio-cultural theory, Cross-cultural research, Qualitative research

## Abstract

There is a growing need for research on culture, cultural differences and cultural effects of globalization in medical education, but these are complex phenomena to investigate. Socio-cultural activity theory seems a useful framework to study cultural complexity, because it matches current views on culture as a dynamic process situated in a social context, and has been valued in diverse fields for yielding rich understandings of complex issues and key factors involved. This paper explains how activity theory can be used in (cross-)cultural medical education research. We discuss activity theory’s theoretical background and principles, and we show how these can be applied to the cultural research practice by discussing the steps involved in a cross-cultural study that we conducted, from formulating research questions to drawing conclusions. We describe how the *activity system*, the unit of analysis in activity theory, can serve as an organizing principle to grasp cultural complexity. We end with reflections on the theoretical and practical use of activity theory for cultural research and note that it is not a shortcut to capture cultural complexity: it is a challenge for researchers to determine the boundaries of their study and to analyze and interpret the dynamics of the activity system.

## Introduction

Medical educationalists increasingly address importance to the role of culture and cultural differences in medical education: an awareness that is fuelled by increasing flows of knowledge, students, teachers, and educational materials, methods and programmes across national, regional and continental borders [1, 2]. While this globalization takes place at a rapid pace in educational practice, research on the effects of globalization, including consequences for national and regional traditions and culture, is largely absent from the field of medical education [1, 3]. Studies that in recent years have responded to the call for (cross-)cultural medical education research highlight how cultural norms and values influence medical education policies and practices [4–10]. While reviewing these studies, the complexity of researching cultural issues surfaces. How to define what culture is, how to meaningfully operationalize research questions, how to empirically investigate cultural values, how to explain their influence in a context where socio-economic, political, historical, institutional and numerous other factors intermingle are some of the struggling questions for cultural researchers. One wonders if the complexity of the cultural phenomenon might be a reason for its under-investigation in the medical education field.

Complexity, however, is not new to this field [11]. Teamwork, inter-professional education, and practice-based learning and assessment of general competencies are but a few examples of medical education areas where researchers deal with complex research contexts. Rather than viewing complexity as an obstacle to clear-cut research, recent perspectives underline that ‘representing complexity well’, including its richness of variation and context, serves the understanding of educational problems and processes as a goal of medical education research [11, 12]. Social perspectives on learning, such as communities of practice and socio-cultural theory in general, have proved useful to investigate complexity for example in teamwork and work-based learning settings [12, 13]. The ability of such approaches to consider the role of the social and cultural environments of education systems is promising to investigate cultural complexity as well, as emphasized by recent cross-cultural research that promotes a socio-cultural conceptualization of medical education research and practice [7, 9].

A particular socio-cultural approach that seems useful to study cultural complexity is activity theory. In diverse areas of medical education activity theory has been valued for its focus on illuminating context issues, determining and analyzing key factors that mediate learning, understanding dynamic forces, challenges and conflicts that underlie educational processes, and providing rich descriptions of ‘what and how it happens’ [12, 14–19], which are all elements that need to be addressed in research on culture, cultural differences, and cultural effects of globalization. The aim of this paper is to explain how activity theory can be used as a theoretical and practical framework to study cultural complexity in medical education. We discuss activity theory’s theoretical background and principles, and we show how activity theory can be applied to the cultural research practice by discussing the research steps involved in a cross-cultural study that we conducted. We end with some reflections on the theoretical and practical use of activity theory for (cross-)cultural research.

## The theory of activity theory

With its roots in the works of the Russian psychologists Vygotsky, Leontiev and Luria, activity theory is grounded in socio-cultural theory [20]. Socio-cultural theory is based on the notion that an individual cannot be viewed as separate from its social and cultural environment [21–23]. Vygotsky [24] emphasized a dialectical perspective of the relationship between man and nature, in which the two continuously affect each other. The process in which individuals are continuously influenced and formed by their environment is defined as *internalization*: humans make constant ‘internal reconstructions of an external operation’ [24]. Simultaneously, they construct and shape their environment, which is defined as *externalization*: a continuous creation of new artifacts which transforms the social and cultural environment [25]. Activity theory is an elaboration of the socio-cultural notion that all human learning and development—and internalization and externalization—takes place in the form of activities [25]. By analyzing these activities, the complexity of learning and development processes can be described, explained and understood. Vygotsky’s perspective of mutually influencing relations between man, the environment and the goal that man acts upon is generally referred to as first-generation activity theory [26]. Engeström elaborated on this perspective and introduced five central principles of activity theory that represent the underlying structure and dynamics of activity, which has been conceptualized as second-generation activity theory [26–28].

**Fig. 1:**
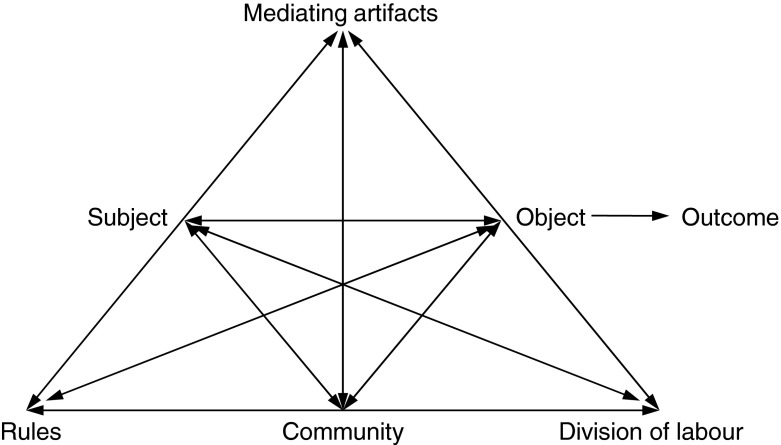
Engeström’s activity system [20]

The first principle states that the unit of analysis is the activity as a whole. A second-generation *activity system* (Fig. 1) represents this unit of analysis, and structures the different components of influence on learning, or on activity in general [20]. The two-sided arrows that run between each of the components indicate that the influence runs both ways. If we take the activity of becoming a doctor as an example, students represent the *subject* of the activity: the group of individuals that carries out the activity. The subject pursues a certain goal—the *object*—such as acquiring knowledge and skills, which leads to the *outcome* of becoming a doctor. During the activity the subject uses tools—*mediating artifacts*—to achieve its goal, e.g. books, lectures and skills training. Furthermore, the subject interacts with the surrounding context: the activity is governed by a set of implicit and explicit *rules*; takes place in a specific social and cultural *community*; and implies certain roles, or a *division of labour*, for those involved [29, 30]. All components of the activity system influence each other in complex interactions. Moreover, in a third generation of activity theory, activity systems as a whole are placed in a network of activity systems that interact with and influence each other [28], e.g. the activity system of becoming a doctor interacts, and sometimes contradicts, with the activity system of providing safe patient care [15]. The second principle of activity theory is *multi*-*voicedness*: an activity involves a collective of interacting individuals and communities who express different interests and views; students from diverse backgrounds and teachers from diverse disciplines might have different ideas and views about ways to become a doctor. The third principle emphasizes the *historicity* of activity: the activity system develops over time and understanding its current form requires knowledge about its past, for example how rules of assessment of clinical skills were developed. The fourth principle focuses on the central role of *contradictions* as a source of change and development of the activity system: the *multi*-*voicedness* of the activity system might lead to tensions, or relations between two components might be contradictory, for example between the *subject* and a newly introduced *mediating artifact* (e.g. a new educational method). Such contradictions could be an incentive for change and transformation of the activity. This leads to the final principle, which refers to the possibility of *expansive transformations*—collective change of the activity, e.g. developing new ways of learning to become a doctor—to result from these contradictions, and from the *internalization* and *externalization* processes involved [28].

In sum, activity theory offers a framework for analyzing the complexity of interactions between individuals and their environment by identifying the components of an activity system, their relations, their different voices, their history, their underlying contradictions, and their transformations. These ingredients might enable cultural researchers to capture the tacit, dynamic and multifaceted concept of culture and its effects, as it is manifested in the activity system. Rather than being a static set of shared values and beliefs, culture is a dynamic process situated in a social context, affected by history, social contradictions, transformations and power relations [31]. Both culture and individuals cannot be viewed, nor researched, separate from their environment. The theory of activity theory supports this view, as does its practice, to which we now turn. A final note on the theory of activity theory is that the above represents a very brief explanation of a complex theory on which many scholars have elaborated. A variety of strands and concepts exist in activity theory, such as theoretical strands focused on understanding, and practical strands focused on designing work-based learning environments [26, 29, 30].

## The practice of activity theory

We conducted a study on the cross-cultural applicability of problem-based learning (PBL) in undergraduate medical education in three medical schools located in the Middle East, East Asia and Western Europe, respectively [8, 9]. We used socio-cultural activity theory as a guiding theoretical and analytical framework. To illuminate its use for cross-cultural research, we discuss the consecutive steps involved in our study. Boxes 1 and 2 provide further details on the methods and results of the cross-cultural case study.

### Formulating research questions

We based our research questions on the socio-cultural notions of *internalization* and *externalization*. We theorized that PBL, being developed in the activity system of Western medical education, carries an inherent set of cultural norms and values that might contradict with learning approaches and attitudes in different parts of the world. While defining PBL as a *mediating artifact* in the *activity system* of becoming a doctor in a particular cultural context, we wondered: a) how do students in this cultural context *internalize* PBL? And b) how do they *externalize* their cultural backgrounds in the PBL process? With these questions we aimed to gain insight into the cross-cultural applicability of PBL. Researchers investigating cultural effects of the globalization of other educational tools, methods and programmes, could formulate similar questions. Also researchers focusing on other aspects and expressions of culture in medical education could benefit from the wide range of activity theory to formulate research questions, in a similar way as activity theory has been used to study diverse medical education issues [12, 14–19]. Though focusing on diverse aspects, a shared aim of these studies is to provide rich descriptions of what and how something happens in a dynamic context of contradictions and transformations.

### Selecting a methodology

To address our research questions we needed to consider not only students and PBL, but all components of the activity systems of becoming a doctor in the three study contexts. In line with activity theory, our methodology needed to enable us to investigate the issue as a whole in its natural local settings and to provide rich data. We selected comparative case study research, based on principles of ethnography, to fulfil these needs. Ethnography typically encourages prolonged engagement in fieldwork to arrive at rich, deep and holistic understandings, using a variety of qualitative data collection methods [32]. Though different methodologies and methods can be used in activity theory [26], methodologies in qualitative research seem most appropriate; particularly ethnography has been used in activity theory research to study complexity in medical education and health care settings [12, 17, 33]. Ethnography’s roots in cultural anthropology, moreover, underline its value for cultural researchers.

### Collecting data

Following from our ethnographic case study approach, we employed various qualitative data collection methods, though we emphasize that activity theory allows many different data collection methods. We will discuss how the methods we chose were thought to grasp the different components of the activity systems and their interactions. More practical information about our methods is reported in Box 1 and elsewhere [8, 9]. During 1 month of fieldwork in each local setting we conducted semi-structured, in-depth interviews with first- and third-year students, tutors, and key staff involved in PBL, we observed PBL sessions in the first and third year, we reviewed documents such as information booklets for students and internal evaluations of PBL, and we took field notes about our experiences, the curriculum, the institute, and the local context outside the institute. By triangulating these diverse data sources we tried to capture the activity system as a whole, though we strongly focused on the activity of PBL, as followed from our research questions.

**Box 1:** Further details on the methods of the cross-cultural case study [8, 9]

*Selecting cases*
We selected the three medical schools based on suggestions of nine medical education experts with ample international experience, who named medical schools that met our criterion of a school in a non-Western setting where PBL had been a substantial teaching method for over five years. An East Asian and a Middle Eastern school were selected from these suggestions and found willing to participate. The Western European school was selected for pragmatic reasons, but met the criterion of using PBL as a substantial teaching method for over 5 years. The Western European and Middle Eastern school had used PBL since their foundation, while the East Asian school had implemented PBL as part of an overall curriculum reform aimed at moving from a teacher-centred to a student-centred curriculum. The reform resulted in a hybrid curriculum that was partly lecture-based and partly PBL
*Selecting participants*
We employed purposive sampling to select interview participants. Male and female students, students from different PBL groups, and students born and raised in the local setting were included. We also included a number of students who had lived and attended school in another country for some time, which we expected to yield richer comparative and multi-voiced information. We included approximately equal numbers of students from the first and the third year to detect differences or transformation. We included tutors from the first and third year with different disciplinary backgrounds. Key staff involved in PBL, such as deans, directors of medical education departments and PBL coordinators, were selected through snowball sampling. Recruitment continued until saturation was achieved. We randomly selected tutorials in the first and third year for our non-participatory observations. In total, 88 interviews and 32 observations were conducted across the cases: 9, 10 and 9 first-year students, 10, 9 and 9 third-year students, 6, 6, and 5 tutors, and 5, 5, and 5 key persons were interviewed in the Middle Eastern, East Asian and Western European case, respectively. Five, 6 and 8 first-year tutorials, and 5, 6 and 2 third-year tutorials, respectively, were observed

We developed an interview scheme that covered the components and development of the activity system according to the five activity theory principles. For example, we asked students and tutors about their ideas about PBL and their practices in PBL to identify how they defined the *activity* of PBL and its components. To identify their social and cultural *communities* we for example asked how the participants would characterize their cultural background, and which norms and values were important to them, to their family and to their society. We considered the *historicity* of the activity system by asking questions about the students’ previous ideas and ways of learning, for example at secondary school, as well as questions for staff members about the history, process and reasons for implementing and applying PBL. We addressed the *multi*-*voicedness* of the activity system by including a diverse range of interview participants, and by asking explicitly about differences in viewpoints among students. We considered *contradictions* in the activity system by asking for example about difficulties that students experienced in PBL, and difficulties that tutors and staff had while implementing and applying PBL. We also included questions about which aspects went smoothly. We addressed the principle of *expansive transformations* by questions about change in students’ beliefs and behaviours of learning, strategies they had developed to deal with PBL, differences between first- and third-year students, and adaptations that were made to the process of PBL since its implementation.

Also during the observations we focused on the activity theory principles, though this was less explicit than in the interviews. We took field notes that described students’ behaviours, and we focused on signs that showed their *internalization* of the PBL model and *externalization* of their cultural learning beliefs and behaviours. Additionally, we filled in an observation sheet that covered aspects of PBL and potential cultural and contextual factors of influence. The practice of activity theory research shows many different ways of collecting data, though their common ground is to yield rich information about the activity system as the unit of analysis.

### Analyzing data

We analyzed our data using template analysis, which is a systematic and hierarchical form of thematic analysis that allows themes to emerge from the data as well as from a theoretical framework [34, 35], in our case activity theory. However, different forms of (thematic) analysis can and have been used in activity theory research [12, 15, 17]. We defined activity theory concepts such as *contradictions*, *tensions* and *transformations* in the activity system as overarching themes and listed prominent themes that emerged from the data as sub-themes. These included cultural norms and values, which we identified based on participants’ answers during the interviews and behaviours observed in fieldwork. We performed a number of iterative coding rounds to identify activity system components and their relations. For each of the three cases we visualized a general activity system of becoming a doctor, with PBL as one of the *mediating artifacts*. Next, we identified components of this system that were of influence on the activity of problem-based learning, and consequently visualized a more detailed activity system of PBL for each of the cases.

We interpreted each of these systems according to the five activity theory principles to understand their dynamics. We looked at important *contradictions, voices,* and *(historic) transformations*, and we analyzed how they came about in the complex interaction between the activity system components. We then compared the activity systems of PBL across the three cases, and explicitly searched for differences and similarities across the cases in terms of components and dynamics of the activity systems. This way we could describe, without dismissing the institutional and socio-cultural contexts, (differences between) how students *internalized* aspects of PBL in three different contexts, as well as (differences between) how their cultural backgrounds were *externalized* in the PBL process in these contexts. In a similar way, researchers of diverse cultural issues might use the structure of the activity system as an organizing principle for the cultural complexity of their research context, without denying or reducing this complexity.

### Reporting results

We reported our results based on two major PBL aspects that we found to contradict with a number of *rules*, *communities* and the *division of labour* in the activity systems across the three cases: self-directed learning and small group discussions [8, 9]. Following the cultural focus of our research questions, we structured the results according to the cultural themes we identified that contradicted with and shaped the self-directed learning and group discussion processes of PBL differently across the three cases through students’ *externalization*. These cultural themes were: group relations, ‘face’ (i.e. concerns not to lose face in the eyes of others), hierarchy, tradition, uncertainty, achievement and competition. In these themes we integrated our findings on how students *internalized* the PBL aspects: how it shaped their self-directed learning skills and discussion and communication skills. Alongside the cultural themes we explicitly focused on other contextual factors of influence on the *internalization* and *externalization* processes, such as language of instruction, systems of assessment, and the nature of students’ secondary education. Box 2 provides a more elaborate summary of the results. We illustrated our results with quotes from interview participants, a visual model of our findings, and tables that specified the roles of contextual factors and degrees of *internalization* and *externalization* [8, 9].

**Box 2:** Further details on the results of the cross-cultural case study [8, 9]

*Externalization: how students shaped PBL across three cultural contexts*
Differences in students’ cultural backgrounds were found to shape group discussions and self-directed learning processes in PBL differently across the three cases. In the Middle Eastern context in particular, students expressed uncertainty and a focus on tradition, which influenced PBL group discussions as students felt anxious to speak up and ask questions. Also, they developed uncertainty-reducing strategies for the self-directed learning element of PBL. In the Middle Eastern and East Asian contexts a strong focus on group relations and face was identified, which impacted on the group discussions as students needed time to establish group relations before feeling comfortable to participate. They were very conscious of maintaining their own and others’ face in front of the group, which influenced discussion dynamics. Acting based on hierarchical relations was another factor of influence in both non-Western contexts in particular. Students preferred to let the tutor speak up, as a source of knowledge and a higher status person, and self-directed learning occurred only to the extent that professors did not cover knowledge, as a higher source of knowledge compared with students’ self-study. In the three contexts, students expressed a strong drive for achievement and competition, though this was least expressed in the Western European context. In the three contexts alike, however, this resulted in decreasing attention to group discussions and self-directed learning if other curriculum aspects were more important for exams and grading, increasing attention if students’ participation substantially counted for grading, and a potential reluctance to share information. Although cultural factors were the study’s main focus, many institutional, organizational, curricular, economic and historical factors were found to mediate the activity of PBL. The shape of PBL in relation to cultural factors was therefore not as straightforward as the above description may suggest. The assessment system, the scope of PBL implementation in the curriculum, the nature of students’ secondary school education, the language of instruction, personality differences between students, and differences between tutors were of major influence on how group discussions and self-directed learning in PBL were shaped
*Internalization: how PBL shaped students across three cultural contexts*
Besides influencing PBL processes, these cultural and other contextual factors also mediated how students were shaped by PBL. Different degrees between the three contexts were found in the development of students’ discussion skills and self-directed learning skills. The hybrid PBL implementation in the East Asian case, for example, implied that students gained a substantial part of their knowledge through lectures, which inhibited the development of self-directed learning skills. Communication skills, however, were encouraged in the East Asian case by students’ focus on achievement and competition coupled with the fact that students were graded for their participation in the PBL sessions. In the Middle Eastern case, students expressed a high level of initial anxiety about PBL, due to cultural factors and the traditional nature of their secondary school education, but they experienced a substantial development of self-directed learning and communication skills because the full scope of the PBL implementation virtually left them no choice. In the Western European case, students expressed lower levels of initial anxiety and few problems with building self-directed learning and communication skills in PBL, due to supportive secondary schooling and cultural factors, but consequently this development was less substantial compared with the gap that Middle Eastern students bridged. Despite these differences, however, PBL was found to gradually shape students across the contexts in a similar direction. Students across the cases were found to build up confidence and comfortableness to participate in group discussions by speaking up, asking questions to peers and tutors, criticizing knowledge and challenging the statements of others. Also, they were found to develop motivation for self-directed learning, an understanding of its purpose, and skills related to searching and finding information, and constructing knowledge
*Conclusions*
In sum, we found that a complex interaction between PBL, students, their cultural backgrounds and other contextual factors determined differences and similarities in the way PBL processes and students were shaped across three cultural contexts. We concluded that, although cultural factors might pose more challenges to applying PBL in non-Western settings, PBL seemed feasible in different cultural contexts. By definition, however, across these contexts PBL processes and outcomes differ according to locally specific, continuously changing activity system dynamics

We did not include the visualized activity systems, which we had developed during data analysis, in the reports of our results, though this is not uncommon in activity theory research [14–16, 18]. In our case we felt the reader would benefit more from a model and structure based on the cultural themes that we found after interpreting and comparing the three cross-cultural activity systems. Cultural researchers could use to their advantage the rich variety in activity theory research of how results can be reported and structured, for example based on *contradictions* that were found [17, 33], or on themes that emerged from the data followed by a discussion of how these related with activity theory [15, 18, 19], or on transformations of *rules*, *communities* and *division of labour* [16], or on a narrative that reflected the dynamics of the activity system [12]. For each research question, creative ways have to be found to represent the cultural complexity that was captured in the collection and analysis of the data well on paper, which can be challenging considering the word limitations of (medical education) journals. Visualizations, either of the activity system or a self-designed conceptual model, illustrative quotes and narratives are examples of useful tools.

### Drawing conclusions

Our findings provided insight into the cross-cultural applicability of PBL by describing a complex interaction between PBL, local students, their cultural backgrounds and the local context, which led to students’ adaptation as well as adaptation of PBL processes across cultural contexts. We concluded that PBL was applicable in diverse cultural contexts, but that its globalization did not postulate uniform processes and outcomes [8, 9]. More important than an answer to the question ‘if PBL could be applied cross-culturally’, was the increased understanding that our findings raised about how PBL was applied cross-culturally, including key influencing factors, which might serve medical educationalists and institutions across cultures that consider adopting PBL or another foreign educational model. As follows from its theory and practice, conclusions from activity theory research move beyond ‘it works’ or ‘it happens’ to ‘how it works’ and ‘how it happens’—illuminating complexity—with possible implications for different settings.

Activity theory’s nature implies that conclusions can be drawn only for the socio-cultural context that was the focus of the study, which is important for cultural researchers to take into account and emphasize. The cultural themes that we have described were based on our data from three medical schools, and although these were located in different cultural areas, we could not generalize our findings to those areas outside the schools. By providing rich descriptions of the cultural and contextual factors involved in a particular case, however, relevant factors, dynamics and implications might be recognized for different settings.

## Reflections on using activity theory to study cultural complexity

Activity theory seems a useful theoretical and practical framework for cultural researchers, because it matches current views on culture as a dynamic process situated in a social context [31], and contains the ingredients to investigate this in practice. The structure of the activity system can serve as an organizing principle for cultural complexity in a way that values this complexity to increase understanding, rather than to dismiss it as ‘noise’ (i.e. as irrelevant or meaningless data that obscure the results’ clarity). Viewing and analyzing the issue under investigation as a whole, and identifying and interconnecting relevant factors involved, can enable researchers to grasp the real-life complexity of local contexts.

Another feature of activity theory that might be valued by cultural researchers is its focus on contradictions between the interacting components of an activity system. Many contradictions are manifested in cultural interactions and cultural effects of globalization, and a focus on these might illuminate cultural processes and explain cultural transformations, such as exemplified by our study on the cross-cultural applicability of PBL. As well as a possible strength, however, the focus on contradictions might be a possible limitation of activity theory, as there is a danger that it overshadows processes that result from congruence, while these are equally important for understanding cultural dynamics. Even though we paid attention to congruent elements during our data collection and analysis, our focus on contradictions was probably stronger as a result of our activity theory perspective. Some studies using activity theory have set a good example by focusing explicitly on both conflict and congruence in research questions, data collection, analysis and results [17], which might be worthwhile for cultural researchers to follow.

A potential difficulty in activity theory, certainly for cultural researchers, is the all-encompassing scope of this perspective. Analyzing an activity as a whole, including a historic perspective and factors from the socio-cultural and institutional communities in which the activity is situated, can be a daunting task. Moreover, the activity itself interacts with surrounding activity systems, implying that the analysis might continue infinitely. Indeed, where does the influence of interconnected activities on cultural values and practices, and vice versa, end? The activity of becoming a doctor, for example, is influenced by numerous other activities, each situated in their own socio-cultural context. In our study we felt challenged to determine the boundaries of the activity systems, including the smaller-scale activity systems of PBL. A challenge for activity theory researchers seems to be to set appropriate boundaries for a study to be feasible, while at the same time do justice to the complexity of a natural setting. We stress that activity theory is not an easy approach or clear-cut recipe to capture complexity: complex phenomena require a complex study approach. Employing an activity theory approach demands a lot from the researchers themselves: the activity system serves as an organizing principle, but it is up to the researchers to identify its components and dynamics, and to make sense from that in relation to their focus and research questions. Also, activity theory does not prescribe specific methodologies or data collection techniques, and researchers are challenged to choose methods that fit their purpose. As described previously, however, qualitative, anthropological methods seem most appropriate for activity theory research on cultural issues.

An interesting addition to the theory and practice of activity theory might be to consider the role of the researcher as an interacting entity with, or a participant in, the activity system (s)he investigates. Researchers are shaped by their socio-cultural and disciplinary backgrounds, and bring this influence, often unconsciously, to the research practice. Especially cultural influences, in the medical field in particular [31], often go unnoticed. Also our study was influenced by researcher bias, as we approached and interpreted the issue with our backgrounds in social and educational sciences and in Western European culture. We took several measures to recognize and address this bias, such as reflexive journaling and member check procedures, but additionally we might have benefitted from placing ourselves explicitly in relation to the activity system we investigated, and analyzing how our socio-cultural and disciplinary backgrounds shaped our understanding of the activity system components and dynamics. Future research on activity theory might elaborate on this issue, which is an essential element that needs to be addressed in cultural research especially.

This paper has explained how activity theory might be used as a theoretical and practical framework to study cultural complexity in medical education, such as cultural effects of globalized educational models. There is an urgent need for cross-cultural research to keep up with the fast-growing developments in global medical education practice [1, 3]. Activity theory offers a lens to capture insights into complex local variations from which global medical education can learn. This moreover extends its use to diverse complex research objects in medical education beside (cross-)cultural research. We emphasize, however, that activity theory is merely one variation of a rich variety of socio-cultural perspectives [36, 37], which offer useful complementary lenses. Similarly as in cultural practice, a rich variation in cultural research approaches serves the goal to represent and understand complexity well.

## Essentials

- Activity theory seems a useful framework for (cross-)cultural research that matches current views on culture as a dynamic process situated in a social context.
- The structure of the activity system can serve as an organizing principle to grasp cultural complexity in a way that values this complexity, rather than to dismiss it as ‘noise’.
- Activity theory’s focus on contradictions is a strength as well as a potential limitation, because (cultural) processes that result from congruence might be ignored.
- Activity theory is not a shortcut to capture (cultural) complexity: it is a challenge for researchers to determine boundaries for the activity they investigate and to analyze and interpret the dynamics of the activity system.
